# Prognostic value of HER2 status in bladder transitional cell carcinoma revealed by both IHC and BDISH techniques

**DOI:** 10.1186/s12885-016-2703-5

**Published:** 2016-08-19

**Authors:** Taoufik Nedjadi, Jaudah Al-Maghrabi, Mourad Assidi, Ashraf Dallol, Heba Al-Kattabi, Adeel Chaudhary, Ahmed Al-Sayyad, Adel Al-Ammari, Adel Abuzenadah, Abdelbaset Buhmeida, Mohammed Al-Qahtani

**Affiliations:** 1 King Fahd Medical Research Center, King Abdulaziz University, PO BOX 80216, Jeddah, 21589 Saudi Arabia; 2 Department of Pathology, Faculty of Medicine, King Abdulaziz University Hospital, Jeddah, Saudi Arabia; 3 Center of Excellence in Genomic Medicine Research, Faculty of Applied Medical Sciences, King Abdulaziz University, PO BOX 80216, Jeddah, 21589 Saudi Arabia; 4 Department of Urology, Faculty of Medicine, King Abdulaziz University Hospital, Jeddah, Saudi Arabia; 5 Department of Urology, King Faisal Specialist Hospital & Research Centre, Jeddah, Saudi Arabia

**Keywords:** *Her2/neu*, IHC, BDISH, Prognosis, Transitional cell carcinoma of bladder

## Abstract

**Background:**

*Her2/neu* is an oncogene that plays an important role in the pathogenesis of many cancer types. In bladder carcinoma (BC), the clinical significance of *Her2/neu* status remains under-investigated and poorly linked to the patients’ clinic-pathological features and survival status. Thus, the current study was conducted to assess *Her2/neu* status in a cohort of patients’ in Saudi Arabia, and to explore its prognostic value in BC.

**Methods:**

A total of 160 consent patients of transitional cell carcinoma (TCC) of bladder were arranged on a tissue microarray (TMA) and stained by immunohistochemistry (IHC) and bright-field dual in situ hybridization (BDISH) methods. The intensity of Her2/neu protein receptor immunostaining was evaluated, correlated to *Her2/neu* gene amplification status in TCC and assessed for potential clinical value by correlation measures.

**Results:**

IHC data demonstrated that Her2/neu protein is expressed in 60 % (2+ and 3+) of our TCC patient’s cohort from Saudi Arabia. *Her2/neu* gene amplification is detected in 25 % by BDISH. There was a strong association between Her2/neu protein levels and lymph node invasion (*p* = 0.04), tumor stage (*p* = 0.002), vascular invasion and borderline significance with distant metastasis (*p* = 0.07). Amplification of *Her2/neu* gene was associated with tumor grade (*p* = 0.03) and poor disease-specific survival (*p* = 0.02), in that, patients with non-amplified *Her2/neu* gene live longer. Interestingly, there was a reasonable concordance rate (71 %) between IHC and BDISH data in the analyzed cohort.

**Conclusion:**

The study showed that 25 % of our patients’ cohort has Her2/neu over-expression. This *Her2/neu* (over-expression/amplification) status was concordant using either IHC or BDISH and significantly associated with disease aggressiveness and poor outcome. These findings suggested a potential impact of anti-Her2 targeted therapy in the treatment of bladder cancer with amplified/overexpressed HER2 that needs further investigation.

## Background

Bladder cancer (BC) is a devastating disease and a leading cause of death worldwide [1]. According to a recently published data, BC is the sixth most common malignancy with an estimated 74,000 new cases and more than 15,000 deaths of bladder cancer in the United States on 2015 [2]. Despite important advances in cancer treatment, the disease continues to pose a big challenge to clinicians due to high recurrence rates, as 50–70 % of new cancer cases recur within 5 years, and a great likelihood to progress to an aggressive, muscle invasive and metastatic forms [3, 4].

The management of BC relies enormously on the patients’ clinico-pathological parameters, such as TNM staging andgrading of the tumor, as indicators of good/poor prognosis. However, these parameters are not sufficient to predict patients’ outcome and worse yet, produce large discrepancies within the same grade/stage. This is mainly related to heterogeneity of bladder cancer cells [5]. Intensive efforts are being made to develop novel molecular tools in order to assist in the detection of the disease at an early stage, enhance stratification of high risk patients and improve clinical management [6, 7]. Although initially promising, most of these tools have not been sufficiently sensitive or specific which necessitate the identification of additional robust biomarkers that would more accurately predict for patient’s prognosis and improve therefore BC surveillance in clinical setting.

The human epidermal growth factor receptor-2 (Her2/neu) is a transmembrane glycoprotein, member of the EGF family of tyrosine kinase receptors encoded by the *Her2/neu* proto-oncogene [8]. In normal circumstances, cells have low Her2 membrane protein content involved in proliferation, differentiation, angiogenesis, and invasion [9] but it increases dramatically in cancer cells. It has been reported that Her2/neu protein is over-expressed in several malignancies especially in gastric (20 %) and breast cancers (up to 30 %) [10–12]. It is well documented that Her2/neu over-expression is associated with poor prognostic in breast cancer. Also, data emerging from clinical trials revealed that the addition of the monoclonal antibody against Her2/neu protein (trastuzumab) to chemotherapy enhanced the overall patients’ survival [13, 14].

A recent analysis of Her2/neu expression status in a large cohort of patients across divers malignancies revealed that bladder cancer exhibited a significant Her2/neu protein over-expression (3+) assessed by immunohistochemistry (IHC) in more than 12 % of patients [15]. In an independent study using 111 BC samples, Bellmunt et al. demonstrated that Her2/neu over-expression was observed in 22 % of the analyzed cohort [16], and could reached in some cases a record ratio of 74 %, as reported elsewhere [17]. This is an interesting finding that indicates that a large proportion of BC patients could benefit from targeted anti- Her2/neu therapies. Moreover, a number of phase II/III clinical trials are currently underway to assess the beneficial effect of anti- Her2/neu and other therapies in urological cancer [18]. The aim of the present study is to determine the status of *Her2/neu* in bladder cancer using two independent methods IHC and BDISH. The level of concordance between the two methods will be evaluated. Correlation between Her2/neu over-expression or gene amplification with patients’ clinico-pathological parameters and the significance of *Her2/neu* as a prognostic biomarker in bladder cancer will be investigated.

## Methods

### Patients

The present series consists of 160 formalin-fixed and paraffin-embedded (FFPE) tissue samples of primary transitional cell carcinoma of bladder cancer retrieved from consent patients’ materials in the archives of the Pathology Department, King Abdulaziz University Hospital (KAUH) (Ethical Approval of KAUH IRB # 149-14). Patients were diagnosed and treated mainly at the Departments of Pathology & Urology, King Abdulaziz University Hospital (KAUH) and King Faisal Specialist Hospital and Research Center (KFSHR), between 1996 and 2012. Only specimens containing more than 80 % tumor cells were used for analysis. The histo-pathological features of the carcinoma specimens were classified according to the TNM classification system. All clinical and pathological data of the patients were collected from the patients’ medical records. The key clinico-pathological data of the patients are shown in Table 1.

**Table 1:** Clinico-pathological characteristics of 160 patients of transitional cell carcinoma of urinary bladder

Clinico-pathological features	Patients number (%)
*Gender*	
Male	133 (83 %)
Female	27 (17 %)
*Age group (years)*	
≤60	70 (44 %)
>60	89 (56 %)
*Lymph node involvement*	
Yes	18 (17 %)
No	86 (83 %)
*Distant metastasis*	
Yes	17 (17 %)
No	83 (83 %)
*Tumor Stage*	
I/II	100 (76 %)
III/IV	32 (24 %)
*Tumor grade*	
Low grade	68 (44 %)
High grade	85 (56 %)
*Vascular invasion*	
Yes	18 (17 %)
No	86 (83 %)
*Recurrence*	
Yes	39 (26 %)
No	113 (74 %)
*Status and end point*	
Alive	107 (68 %)
Died	50 (32 %)

### Tissue microarray

Based on tissue microarray technique (TMA) as previously described [19], we successfully transferred approximately 160 blocks of bladder cancer to construct TMA slides for evaluating the expression pattern of Her2/neu status. Moreover, we also validated the TMA technique in an integrative and comprehensive approach with IHC for protein profiling of Her2/neu protein and BDISH for Her2/neu gene profiling in bladder cancer samples. The TMA technique seems to us as a feasible, useful and multipurpose platform.

### Immunohistochemistry (IHC)

Overexpression of Her2/neu protein was detected automatically by IHC. The FDA-approved Her2/neu IHC assay using PATHWAY Her2/neu rabbit monoclonal antibody (clone 4B5; Ventana) was performed with iView DAB Detection Kit (Ventana) on a BenchMark XT automated staining system (Ventana). A protocol was established so that the entire assay procedure consisting of deparaffinization with EZ Prep (Ventana) at 75 °C, heat pretreatment in Cell Conditioning 1 (CC1; Ventana) using “conditioner CC1 8 min,Mild CC1 30 min” for antigen retrieval at 100 °C, and then incubation with the anti- Her2/neu primary antibody for 16 min at 37 °C. The slides were counterstained with Hematoxylin II (Ventana) for 4 min and Bluing Reagent (Ventana) for 4 min. At the completion of the run, the slides were removed from the automated slide stainer. Following the staining step, the slides having residual buffer and liquid coverslip solution on them were rinsed first with a mild detergent followed by water until complete removal of the soap. The slides were then immersed into successive alcohol buffer at different concentrations (70 %, 95 %, 100 %) for 3 min to each. Then one drop of Tissue-Tek glas mounting medium was applied onto a slide and covered with the glass coverslip.

### Bright Field Double In Situ Hybridization (BDISH)

*Her2/neu* and Chromosome 17 probes were detected using two colors chromogenic in situ hybridization (ISH) in formalin-fixed, paraffin-embedded human bladder cancer tissue specimens following staining on VENTANA BenchMark XT automated slide stainer, by light microscopy. The INFORM *Her2/neu* Dual ISH DNA Probe Cocktail contains a *Her2/neu* probe (labeled with the hapten dinitrophenyl or DNP) and a Chromosome 17 centromere probe (labeled with the hapten digoxigenin or DIG) formulated with human placental blocking DNA in a formamide-based buffer. The probes were designed to detect amplification of the *Her2/neu* gene in solid tumors. A protocol was established so that the entire assay procedure consisting of baking for 30 min, deparaffinization with EZ Prep (Ventana) at 75 °C, cell conditioning 2 (CC2; Ventana) using “Mild CC2 cycle 8 min, standard CC2 cycle 12 min, extended cycle 8 min” followed by protease digestion with ISH protease 3 (ventana) for 16 min at 37 °C. The genomic DNA in tissue sections and the nick-translated HER2 and CEN17 probes were denatured by heat treatment for 20 min at 80 °C followed by a hybridization step for 6 h at 44 °C. After that, 3 stringency wash steps were performed at 72 °C with 2× SCC (Ventana). For HER2 gene detection, the slides were incubated with a rabbit anti-DNP antibody for 20 min and then with HRP-conjugated anti-rabbit antibody for 16 min at 37 °C. The *Her2/neu* BDISH signal was detected as metallic silver deposits with silver acetate, hydroquinone, and hydrogen peroxide for 4 min at 37 °C. For CEN17 detection, the slides were incubated with a mouse anti-DIG antibody for 20 min and then with an alkaline phosphatase (AP)-conjugated anti-mouse antibody for 24 min at 37 °C. The CEN17 BISH signal was developed as red dot staining with fast red and naphthol phosphate for 8 min. Finally, the slides were counterstained with Hematoxylin II for 8 min and with Bluing Reagent for 4 min. At the completion of the run, stained slides having residual buffer and liquid coverslip solution were rinsed in a mild detergent to remove the coverslip solution, then rinsed with water only until soap was completely removed and finally dried at 45 °C for 15 min. Slides were thereafter transferred into xylene bath for approximately 30 s then coverslips were applied by the Tissue-Tek Film Cover slipper.

### Scoring system of Her2 gene/protein amplification/expression status

In order to assess both Her2/neu protein expression pattern and corresponding gene amplification status in bladder cancer, we have used the same scoring system that has already been implemented for breast cancer following the current ASCO/CAP guidelines [20]. The scoring method for Her2 protein expression is based on the cell membrane staining pattern. Her2/neu testing results by IHC fall into 3 categories: positive, equivocal, and negative. Each of these results triggers different patient management. Cancer samples with equivocal IHC were validated using BDISH to assess the HER2 gene amplification status. The interpretation for *Her2/neu* BDISH testing (*Her2/neu*/Chr17 ratio) is according to (ASCO/CAP) scoring systems. BDISH staining results in visualization via light microscopy in which *Her2/neu* appears as discrete black signals (SISH) and Chr17 as red signals (Red ISH) in nuclei of cells. The Scoring Algorithm for the *Her2/neu* Dual ISH DNA Probe Cocktail is as following; non-amplified (*Her2/neu*/CEP17 ratio <1.8), equivocal (HER2/CEP17 ratio 1.8–2.2), and amplified (*Her2/neu* /CEP17 ratio >2.2). However, according to new regulation of ASCO/CAP guidelines for ISH technique if the result is equivocal (*Her2/neu*/CEP17 ratio is 1.8–2.2), you have to count extra 20 cells for both *Her2/neu* and CEP17 and calculate the ratio once again. If the ratio is below <2, non-amplified; and if ratio is above >2, it is amplified.

### Control samples

For quality control purposes, we used *Her2/neu* Dual ISH 3-in-1 Xenograft of breast cancer slides provided by the company (Roche Diagnostics GmbH, Mannheim, Germany) representing the *Her2/neu* gene status in its 3 different categories; amplified (+3), equivocal (+2) and non-amplified (0, +1), respectively. We stained these xenograft tumor slides throughout the same run for staining bladder cancer samples in both IHC and BDISH experiments.

### Statistical analysis

Statistical analyses were performed using the SPSS® (IMB NY, USA) software packages (PASW Statistics for Windows, version 19). Frequency tables were analyzed using the Chi-square test, with likelihood ratio (LR) or Fischer’s exact test to assess the significance of the correlation between the categorical variables. Odds Ratios (OR) and their 95 % Confidence Intervals (95 % CI) were calculated where appropriate, using the exact method. Univariate survival analysis for the outcome measure (DSS, DFS) was based on Kaplan-Meier method, with log-rank (Mantel-Cox) comparison test. In all tests, the values *p* < 0.05 were considered as statistically significant.

## Results

### Expression patterns of Her2/neu protein profiling & amplification status of Her2/neu gene in transitional cell carcinoma of urinary bladder

**Fig. 1:**
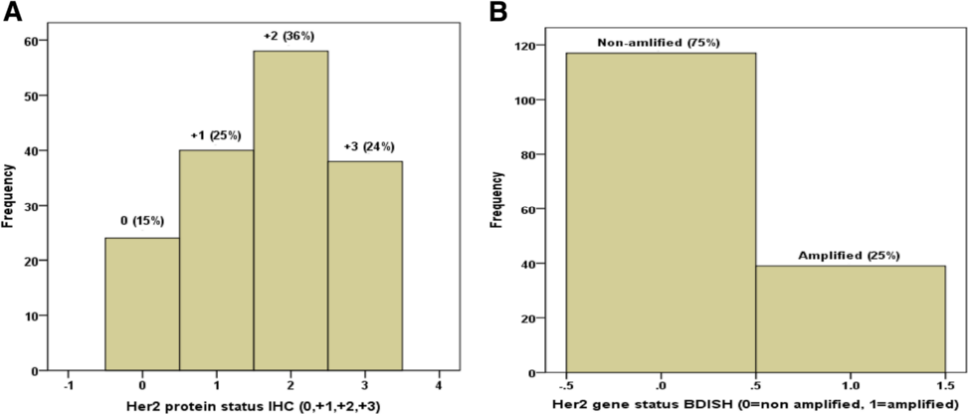
Histograms showed the frequency of expression patterns of Her2/neu protein receptors in 160 of bladder cancer by IHC (**a**) with corresponding amplification status of her2/neu gene of the same samples by using BDISH technique (**b**)

**Fig. 2:**
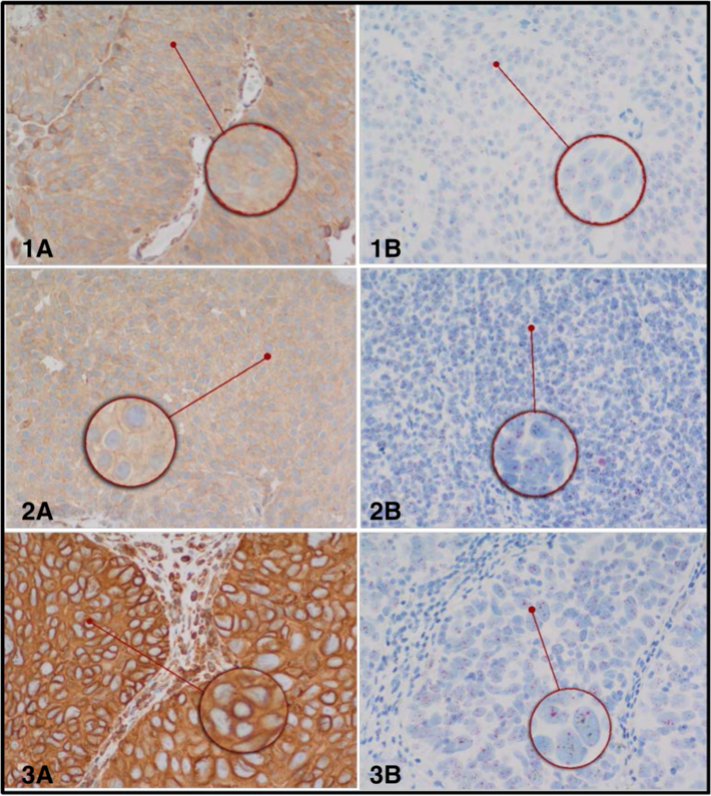
Validation and concordance rate of both IHC (A1-3) and corresponding BDISH (B1-3) of TMA spots for Her2/neu protein expression and *Her2/neu* gene amplification patterns, respectively. Figures (*1a-1b*) represented 1+ membranous expression patterns (negative) of Her2/neu protein (*1a*) and Non-amplified *Her2/neu* gene status (*1b*). Figures (*2a-2b*) represented 2+ membranous expression patterns (borderline) of Her2/neu protein (*2a*) and amplified *Her2/neu* gene status (*2b*). While, figures (*3a-3b*) represented 3+ membranous expression patterns (high) of Her2/neu protein (*3a*) and corresponding amplified *Her2/neu* gene status (*3b*) in TCC of urinary bladder. Magnification is X40

The staining patterns and prevalence rate of Her2/neu protein profile and amplification status of *Her2/neu* gene respectively, are illustrated in Fig. 1. The expression of Her2/neu protein is mainly membranous. The frequencies of expression patterns of Her2/neu protein receptors in 160 of bladder cancer samples evaluated by IHC technique were: no expression (negative, 15 %), weak expression (+1, 25 %), moderate expression (+2, 36 %) and strong expression patterns (+3, 24 %), respectively (Fig. 2. A1-3), while the corresponding frequencies of amplification status of *Her2/neu* gene evaluated by BDISH technique were: non-amplified *Her2/neu* gene status (75 %) and amplified Her2/neu gene status (25 %), respectively (Fig. 2. B1-3).

### Concordance rate between IHC and BDISH for evaluating Her2/neu protein/gene status in transitional cell carcinoma of urinary Bladder

Data showed that the two techniques have a reasonable concordance rate in evaluating the *Her2/neu* status in bladder cancer (Table 2). The concordance rate was estimated in 156 samples. Interestingly, 27/38 (71 %) of bladder cancer samples which, showed +3 over-expression patterns of Her2/neu protein have amplification (> 2) of their corresponding *Her2/neu* gene while only 29 % (11/38) of over-expressed Her2/neu proteins showed non-amplified (< 2) *Her2/neu* gene, respectively. Surprisingly, all cases of low expression patterns of Her2/neu protein profile (0/+1) 60/60 (100 %) showed non-amplification (< 2) of corresponding *Her2/neu* gene, i.e. also that no sample 0/60 (0 %) of low expression (0/+1) samples showed amplification (> 2) of corresponding *Her2/neu* gene. While in cases of borderline/equivocal (+2) expression pattern of Her2/neu protein, attractively, just about 46/58 (79 %) of samples showed non-amplification (< 2) of corresponding *Her2/neu* gene and 12/58 (21 %) of samples showed amplified (> 2) correspondent *Her2/neu* gene, respectively (*p* < 0.005).

**Table 2:** Concordance rate between IHC and BDISH techniques for evaluating Her2/neu protein/*gene* status in transitional cell carcinoma of urinary bladder (*p* < 0.005)

		*Her2/neu* gene status (BDISH)		
		Non-Amplified (≤2)	Amplified (>2)	Total
Her2/neu protein expression patterns (IHC)	Weak Expression (0,1+)	60 (100 %)	0 (0 %)	60
	Borderline/equivocal Expression (2+)	46 (79 %)	12 (21 %)	58
	High Expression (3+)	11 (29 %)	27 (71 %)	38
	Total	117	39	156

### Correlation of IHC Her2/neu protein expression and BDISH Her2/neu gene status with clinico-pathological features

Our data showed that both age and gender had no significant relationship with the expression pattern of Her2/neu protein (Table 3). However, the involvement of lymph nodes was significantly associated with more Her2/neu protein expression pattern (*p* < 0.04). Regarding the stage of the tumor, there was a highly significant correlation between expression patterns of Her2/neu protein and stage of the disease. In fact, high stage tumors expressed more Her2/neu protein receptors compared to those of low stage (*p* < 0.002). Interestingly, the same trend was also observed with vascular invasion status where tumors with vascular invasion showed more expressed Her2/neu protein pattern profile as compared to tumors with no vascular invasion (*p* < 0.01). However, a correlation of borderline significance was observed between tumors with distant metastasis as contrasted to those with no metastasis (*p* < 0.07). Moreover, the study demonstrated that higher tumor grade was associated with amplified *Her2/neu* gene status as compared to low grade tumors (*p* < 0.03, data not shown).

**Table 3:** Correlation between Her2/neu protein expression patterns and clinico-pathological features of transitional cell carcinoma of urinary bladder

Features	Number of cases (%)	Her2/Neu protein expression patterns^a^ (%)			*p* value
		(0,1+)	(2+)	(3+)	
Gender					0.19
Male	133 (83 %)	49 (37 %)	51 (38 %)	33 (25 %)	
Female	27 (17 %)	15 (55 %)	7 (26 %)	5 (19 %)	
Age group (years)					0.92
≤60	70 (44 %)	27 (39 %)	26 (37 %)	17 (24 %)	
>60	89 (56 %)	37 (42 %)	31 (35 %)	21 (24 %)	
Lymph node involvement					0.04
Yes	18 (17 %)	9 (50 %)	2 (11 %)	7 (39 %)	
No	86 (83 %)	33 (39 %)	35 (40 %)	18 (21 %)	
Distant metastasis					0.07
Yes	17 (17 %)	9 (53 %)	2 (12 %)	6 (35 %)	
No	83 (83 %)	31 (37 %)	34 (41 %)	18 (22 %)	
Tumor Stage					0.002
I/II	100 (76 %)	37 (37 %)	43 (43 %)	20 (20 %)	
III/IV	32 (24 %)	18 (56 %)	3 (10 %)	11 (34 %)	
Tumor grade					0.62
Low grade	68 (44 %)	27 (40 %)	27 (40 %)	14 (20 %)	
High grade	85 (56 %)	35 (41 %)	28 (33 %)	22 (26 %)	
Vascular invasion					0.01
Yes	18 (17 %)	10 (55 %)	1 (6 %)	7 (39 %)	
No	86 (83 %)	31 (36 %)	36 (42 %)	19 (22 %)	
Recurrence					0.61
Yes	39 (26 %)	14 (36 %)	15 (38 %)	10 (26 %)	
No	113 (74 %)	47 (42 %)	40 (35 %)	26 (23 %)	
Status and end point					0.59
Alive	107 (68 %)	41 (38 %)	41 (38 %)	25 (24 %)	
Died	50 (32 %)	22 (44 %)	15 (30 %)	13 (26 %)	

^a^(0,1+) = no expression and weak, (2+) = borderline and (3+) = high Her2/neu protein expression patterns

### Correlation of IHC Her2/neu protein expression and BDISH Her2/neu gene status with survival outcome

**Fig. 3:**
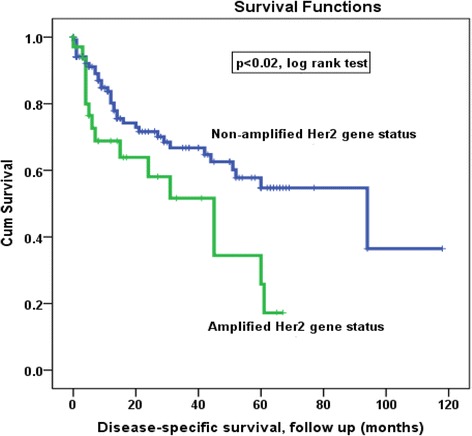
Kaplan Meier analysis of *Her2/neu* gene status in bladder cancer. Disease-specific Survival (DSS) outcome for patients according to their *Her2/neu* gene amplification status (*p* < 0.02, log-rank test)

The survival data were available for 155 patients. Kaplan–Meier survival analysis showed that there was a significant (*p* < 0.02, log rank) difference in DSS between patients with non-amplified *Her2/neu* gene status tumors who living significantly longer (longer DSS) as compared with those with amplified *Her2/neu* gene status tumors (Fig. 3). In other words, the study showed that at 60 months (5 years) follow up time, about 80 % of patients with amplified *Her2/nue* gene were died of disease as compared to only 42 % of their counterparts with non-amplified *Her2/neu*. By using the IHC score, the survival analysis showed a trend/tendency of high recurrence rate of cancer patients with high Her2 protein expression pattern as compared to patients who have cancer with low Her2 protein expression pattern. However this correlation was not statistically significant (*p* < 0.1, log rank, data not shown).

## Discussion

Bladder cancer is the ninth most common and a leading cause of cancer death worldwide [1]. High recurrence rate and heterogeneous features of urothelial bladder cancers are the major hurdles that hamper a suitable clinical management [21]. The latter depends enormously on several clinico-pathological factors such as tumor grade, stage and lymph node invasion [22]. Hence there is an urgent need to develop new diagnostic and therapeutic modalities to meet clinical needs for the management of bladder cancer. Recent advances in cancer research have improved our understanding of the biological features of the disease and unveil relevant biomarkers that are currently used in cancer stratification, screening, diagnosis and prognosis [23]. In the last decade, human epidermal growth factor receptor 2 (Her2/neu) has emerged as a potential biomarker in many cancer types especially breast, colorectal and gastric cancers [14, 24–26]. Over-expression of Her2/neu protein and gene amplification is associated with increased tumor growth and poor prognosis and targeted Her2/neu therapy has proven to be promising in breast cancer treatment [13]. However, the clinical relevance of Her2/neu in bladder cancer remains ambiguous and under-investigated. This fact has strongly urged us to analyze the expression patterns of *Her2/neu* status using two independent experimental techniques to assess its prognostic and/or predictive value in our cohort of bladder cancer patients, therefore better clinical management scenarios will be foreseen for them.

In the present study, we used a cohort of 160 consent patients with TCC of bladder cancer along with their full set of clinico-pathological data. IHC results showed that that Her2/neu protein was expressed in 60 % of the analyzed samples. The percentage of the Her2/neu protein over-expressed (3+) was observed in 24 % of patients. Previous published reports revealed that over-expression of Her2/neu protein is highly variable in bladder cancer ranging from 9.2 to 71 % [27–29]. This variability could be attributed to several factors such as sample size, detection method used, scoring criteria and ethnic origin [16]. However, the most important finding in the current study was that expression of Her2/neu correlated significantly with tumor stage, vascular invasion, lymph node invasion and distant metastasis. Our observation is consistent with previous findings where Her2/neu status correlated significantly with lymph node metastases from urothelial bladder cancer compared to the primary tumors [30, 31]. This observation indicates that Her2/neu is a valuable biomarker and/or effector associated to the metastatic potential of tumor cells and provide merit to exploring targeted Her2/neu therapy in patients with metastatic bladder cancer [16, 32].

Interestingly, high correlation between *Her2/neu* gene amplification and tumor grade was observed (*p* = 0.03). Increased amplification of *Her2/neu* was more common in higher histological grades than lower ones in several cancers including breast, bladder and osteosarcoma [33–35]. Similar correlations between *Her2/neu* status with high-grade and advanced stages (III/IV) were also reported in bladder cancer supporting more aggressive and proliferative diseases with these patients [36].

In an independent approach, we carried out BDISH to investigate the status of *Her2/neu* gene in order to validate our IHC 2+ group and assess the concordance between the two techniques. Despite some discrepancies regarding the level of concordance between IHC and FISH methods [37–39], so often both techniques consolidate each other and generate similar results [27, 40, 41]. Despite that IHC is more cost-effective and recommended by some studies [42], it has been reported that assessing *Her2/neu* gene amplification using in situ hybridization technique is a more reliable and accurate method to evaluate *Her2/neu* status in breast cancer patients [36, 43]. Here we report that *Her2/neu* is amplified in 25 % of the analyzed cohort which is in line with IHC data. Although a reasonable concordance rate for *Her2/neu* testing by IHC and BDISH was achieved in our cohort, higher concordance between these two platforms were also previously reported in urothelial carcinoma [44]. This rate is below the threshold mandated by the new ASCO–CAP guidelines [45]. However, the use of the emerging digital droplet PCR as a new promising technology which may help improving the accuracy of *Her2/neu* copy number variation and reinforce these two FDA-approved approaches for better clinical outcomes [46].

Kaplan Meier survival curve revealed that amplification of *Her2/neu* gene is significantly associated with a poor prognosis and shorter overall survival (Fig. 3). However, patients with non-amplified *Her2/neu* gene were associated with prolonged DSS suggesting *Her2/neu* as an independent prognosticator of overall survival in bladder cancer (*p* = 0.02). This association was previously examined in many cancer types [12, 36, 47, 48]. The enhancement of cancer cell proliferation, motility, invasion and metastasis are possible factors that support such poor prognostic impact of *Her2/neu* on the overall survival in bladder cancer patients [49–51]. Additionally, previous findings reported an interesting impact of anti-Her2 targeted therapy (Trastuzumab) in the treatment of bladder cancer with amplified/overexpressed HER2. In fact, 70 % of bladder cancer patients with amplified HER2 were shown to respond completely or partially but safely to Trastuzumab treatment [52, 53]. Additional studies using larger patients’ cohorts and randomized trials are required to validate these urothelial bladder cancer survival outcomes results and to investigate further the anti-HER2 targeted therapy in Her2/neu overexpression patients in clinical trial setting.

## Conclusion

Our results showed a reasonable concordance rate between IHC and BDISH supporting that both approaches can be used to assess the *Her2/neu* status. The discrepancies between both approaches are expected since they are affected by the experimental context and the pre-analytical conditions. The use of digital droplet PCR could be a good alternative for validation of these two FDA-approved approaches given its high accuracy, sensitivity and cost-effectiveness.

We reported herein and for the first time in Saudi Arabia that 24 % of our patients’ cohort have Her2/neu over-expression. Although this *Her2/neu* (over-expression/ amplification) status reported herein requires further validation using larger cohorts of patients, it was significantly associated with disease aggressiveness and poor outcome. Our results together with previous findings provide new insight into the role of Her2*/neu* in TCC patients who might benefit from anti-*Her2/neu* targeted therapy in clinical trial setting especially those with locally advanced and metastatic Her2*/neu* amplified bladder cancer.

## Abbreviations

ASCO: American society of clinical oncology
BC: Bladder cancer
BDISH: Bright field double in situ hybridization
CAP: College of American pathologists
DFS: Disease free survival
DSS: Disease specific survival
FDS: Food and drug authority
Her2: Human epidermal growth factor receptor 2
IHC: Immunohistochemistry
TCC: Transitional cell carcinoma
TMA: Tissue microarray
TNM: Tumor, node and metastasis
